# A method for reconstituting the motility of membrane-bound myosin on the surface of the cell-sized W/O droplet

**DOI:** 10.1016/j.mex.2025.103755

**Published:** 2025-12-09

**Authors:** Yusei Sato, Rieko Sumiyoshi, Masahito Hayashi, Masahiko Yamagishi, Junichiro Yajima

**Affiliations:** a Department of Life Sciences, Graduate School of Arts and Sciences, The University of Tokyo, 3-8-1 Komaba, Meguro-ku, Tokyo 153-8902, Japan; b Institute for Extra-cutting-edge Science and Technology Avant-garde Research (X-star), Japan Agency for Marine-Earth Science and Technology (JAMSTEC), 2-15 Natsushima-cho, Yokosuka, Kanagawa 237-0061, Japan; c Department of Biotechnology and Life Science, Tokyo University of Agriculture and Technology, 2-24-16 Naka-cho, Koganei, Tokyo 184-8588, Japan; d Komaba Institute for Science, The University of Tokyo, 3-8-1 Komaba, Meguro-ku, Tokyo 153-8902, Japan; e Research Center for Complex Systems Biology, Universal Biology Institute, The University of Tokyo, 3-8-1 Komaba, Meguro-ku, Tokyo 153-8902, Japan

**Keywords:** Myosin, Actin, Artificial cell, Motility, Cell membrane, Phospholipid

## Abstract

Membrane-bound myosin generates force through interactions with the cytoskeletal actin filament beneath the cell membrane and constitutes the mechanical basis for living cells. Myosin ID, a membrane-bound myosin, drives the gliding motion of actin filaments and binds to phospholipids in the lipid membrane of a living cell. Here, we describe the *in-droplet* actin filament gliding assay, a method that reconstitutes the motility of actomyosin that dynamically interacts with lipid membranes within water-in-oil (W/O) droplets, which mimic the confined geometry of the intracellular environment. Our method enables quantification of the gliding velocity of actin filaments driven by myosin ID on the inner surface of W/O droplets surrounded by a phospholipid membrane. The *in-droplet* actin filament gliding assay provides a valuable platform for reconstituting the motile properties of other membrane-bound myosins on membrane surfaces in confined spaces and for analyzing the dynamics of actomyosin networks. The main features and applications of this method are as follows:

• Reconstitution of actin filament gliding driven by membrane-bound myosin ID within confined water-in-oil droplets.
• Quantitative evaluation of actomyosin dynamics on the inner surface of the lipid membrane within water-in-oil-droplets.
• Broadly applicable assay platform for studying the motile properties of membrane-associated myosin families.

## Graphical abstract

**Figure:**
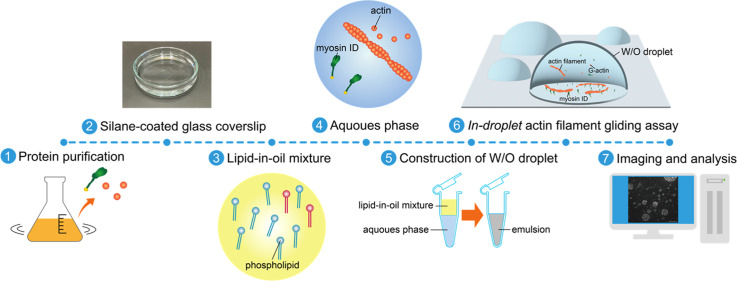

**Specifications table**

**Table:** 

**Subject area**	Biochemistry, Genetics and Molecular Biology
**More specific subject area**	Motility assay for motor proteins
**Name of your method**	*In-droplet* actin filament gliding assay
**Name and reference of original method**	*Yusei Sato, Rieko Sumiyoshi, Masahiko Yamagishi, Takeshi Haraguchi, Kyohei Matsuda, Suguru Sato, Kohji Ito, Junichiro Yajima, “Reconstructing the Motility Driven by Membrane-Bound Myosin on the Inner Surface of Cell-Sized Droplets”, Langmuir, 41 (2025), 10,077–10,084,*https://doi.org/10.1021/acs.langmuir.4c04123
**Resource availability**	N/A

## Background

Myosin molecular motor and the actin cytoskeleton constitute the mechanical basis of fundamental cellular processes, playing essential roles in intracellular transport, cell division, cell migration, and the regulation of cell morphology. Myosin generates force and moves along actin filaments through an ATP-hydrolyzing reaction cycle. To investigate the molecular mechanisms underlying the motility of myosin and actin filament, a widely used *in vitro* experimental technique known as the gliding assay has been employed since the 1980s [1]. This assay reconstitutes cytoskeletal motility by immobilizing motor proteins on a rigid substrate, allowing quantitative evaluation of the gliding velocity of actin filaments across a myosin-coated surface [2].

To study the physiological interactions between myosin and the actin cytoskeleton *in vitro*, it is beneficial to construct a geometry that mimics the intracellular environment. Actin filament gliding assays have been performed on supported lipid bilayers, which are planar phospholipid bilayers formed on glass surfaces that mimic the fluidity of the cell membrane. These systems have successfully reconstituted the motility of myosin and actin filaments on biologically relevant membranes [[3], [4], [5]]. Another valuable experimental platform is the water-in-oil (W/O) emulsion droplet, which consists of an aqueous phase containing biomolecules and an oil phase containing phospholipids. W/O droplets provide both a fluid lipid membrane surface and an encapsulated geometry that mimics the confined intracellular environment. This system may enable the reconstruction of physiological interactions between proteins and cell membranes under confined geometry [[6], [7], [8]].

We describe a method, the *in-droplet* actin filament gliding assay, that enables gliding assays on the inner aqueous surface of a W/O droplet. We perform this assay using myosin ID, a member of the single-headed myosin I family. Myosin ID consists of two major regions: (i) an N-terminal motor domain that includes the actin-binding site and the ATP-binding site, and (ii) a C-terminal tail domain that contains a pleckstrin homology (pH) domain which binds specifically to phosphatidylinositol 4,5-bisphosphate (PI(4,5)P_2_) in the cell membrane [9,10]. Myosin ID exhibits highly dynamic interactions with the PI(4,5)P₂-containing phospholipid membrane via its pH domain in the tail region [11]. Through this interaction, myosin ID links cytoskeletal actin filaments to the cell membrane and plays essential roles in various physiological processes [12,13].

In our method, myosin ID and G-actin are encapsulated within a W/O droplet containing PI(4,5)P₂. This setup allows the reconstitution of the dynamic interaction between myosin ID and the PI(4,5)P₂-containing phospholipid membrane, as well as the gliding motion of polymerized actin filaments driven by myosin ID. Our technique provides a useful platform for evaluating the dynamics of actomyosin networks at membrane interfaces in confined spaces and is readily applicable to studies of other membrane-associated myosin families.

## Method details

### Preparing biological samples

Myosin ID was expressed using the High Five™ cell culture (Invitrogen) and baculovirus system. A detailed description of the baculovirus expression procedure is beyond the scope of this article. Myosin ID was purified following a previously established protocol [14], the details of which are also omitted here. Briefly, we describe the preparation of recombinant *Drosophila* myosin ID with a tag for anchoring the motor in the gliding assay. cDNA constructs encoding full-length myosin ID with a C-terminal His tag and Myc tag, and an N-terminal FLAG tag were inserted into a pFastBac vector (Invitrogen) to generate the baculovirus transfer vector. Myosin ID was co-expressed with calmodulin in High Five™ cell cultures and purified using anti-FLAG M2 affinity resin (Sigma-Aldrich, USA). Calmodulin was expressed in *Escherichia coli* strain BL21 (DE3) and purified using the trichloroacetic acid precipitation method, followed by affinity chromatography with Phenyl Sepharose CL-4B (GE Healthcare, USA). Purified myosin ID and calmodulin were stored at −80 °C. G-actin was purified from acetone powder using a standard protocol [15] and stored in liquid nitrogen.

### Preparing silane-coated glass coverslips

In our method, the *in-droplet* actin filament gliding assay, glass coverslips are used as substrates for observing water-in-oil (W/O) droplets containing myosin ID and actin filaments. Hydrophobization of the coverslip surface with dimethyldichlorosilane prevents nonspecific adsorption of the phospholipid membrane of the W/O droplet to the glass surface. Uniform coating is important: incomplete hydrophobization may lead to droplet flattening or rupture. Without proper hydrophobization, actin filament gliding may stall and fail within a short time. Therefore, this step is critical for stable observation. All processes in this section are conducted at room temperature.

Materials:

• 24 mm × 36 mm glass coverslips (#C024361, Matsunami Glass Industry, Japan)

Chemicals and reagents:

• Silanization solution I (#85126, Sigma-Aldrich, USA)
• Acetone (#012–00343, Fujifilm Wako Pure Chemical, Japan)
• Ethanol (#057–00451, Fujifilm Wako Pure Chemical, Japan)
• Potassium hydroxide (KOH) (#168–21815, Fujifilm Wako Pure Chemical, Japan)
• Ultrapure water (Milli-Q) (#IQ 7003, Merck, Germany)

Equipment:

• KOH-resistant ceramic or PTFE coverslip rack
• Glass beaker (300 mL)
• Petri dish (7 cm)
• Sonicator (#2510, Branson)
• Vacuum desiccator
• Titanium dioxide-coated tweezers
• Nitrile gloves

Step-by-step protocol:

1. Glass coverslips were placed on a coverslip rack using titanium dioxide-coated tweezers (Fig. 1a).

**Fig. 1:**
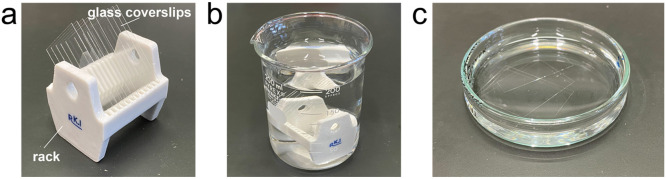
**Silane coating procedure for glass coverslips.** (**a**) Glass coverslips and glass rack. (**b**) Glass rack in 300 mL beaker. (**c**) Glass coverslips within silanization solution I in a petri dish.
2. To remove dust and residual coating on the glass surface, the rack with coverslips was immersed in 5 M KOH solution in a 300 mL beaker and left overnight (Fig. 1b).
3. The coverslips were then transferred to a fresh 300 mL beaker and thoroughly washed with ultrapure water by repeatedly replacing the water.
4. The coverslips were transferred to a new 300 mL beaker containing 200 mL of acetone to remove water.
5. The beaker was placed in a sonicator and sonicated for 5 min.
6. The coverslips were then immersed in silanization solution I for 1 hour in a Petri dish. This step was performed in a fume hood (Fig. 1c).
7. After silanization, the coverslips were returned to the coverslip rack.
8. The rack was placed in a new 300 mL beaker containing 200 mL of acetone and sonicated for 5 min to remove silanization solution.
9. The coverslips were transferred to another fresh 300 mL beaker with 200 mL of acetone and sonicated again for 5 min.
10. The coverslips were then transferred to a new 300 mL beaker containing 200 mL of ethanol and sonicated for 5 min.
11. The coverslips were transferred to a new 300 mL beaker containing 200 mL of ultrapure water and thoroughly washed by replacing the ultrapure water several times.
12. Finally, the silane-coated coverslips were dried and stored in a clean vacuum desiccator to maintain the hydrophobicity of the glass surface. Silane-coated coverslips can be used for experiments for up to one month.

Validation:

The hydrophobization of the coverslip surface is critical for stable observation of W/O droplets. In practice, successful coating is confirmed by placing test W/O droplets on the coverslip and observing whether the droplets remain spherical without flattening or rupturing. If droplets show abnormal behavior, the coverslip is considered inadequately coated and should be re-coated. Ideally, contact angle measurements could also be performed to quantitatively assess the efficiency of hydrophobization [16].  

Troubleshooting:

- If the coverslip appears white or is not sufficiently hydrophobic after this procedure, repeat the coating using new coverslips and a fresh KOH solution.

## Preparing the lipid-in-oil mixture

The lipid-in-oil mixture forms the outer membrane layer of the W/O droplet. The interactions between motor proteins encapsulated in the aqueous phase of the W/O droplet and the membrane surface are regulated by the type of myosin tag and the phospholipid composition of the membrane. In this method, DOPC is used as the main component of the lipid membrane and PI(4,5)P₂ acts as a linker that specifically interacts with the pH domain of myosin ID in the aqueous phase. Proper preparation of the lipid-in-oil mixture ensures the formation of a stable monolayer at the oil–water interface, with the lipid hydrophilic headgroups oriented toward the aqueous phase and hydrophobic tails embedded in the oil. This arrangement is essential for maintaining the structural integrity of W/O droplets and enabling reliable interactions between the membrane and myosin ID. Incomplete dissolution of lipids or prolonged heating of the oil phase can impair monolayer formation and droplet stability. Users should visually inspect the mixture to confirm uniformity and avoid discoloration, which can indicate lipid degradation. Uniform lipid distribution and avoidance of overheating are critical to prevent droplet flattening, rupture, or experimental failure.

Chemicals and Reagents:

• DOPC powder (#850375P, Avanti Polar Lipids, USA)
• PI(4,5)P_2_ powder (#850155P, Avanti Polar Lipids, USA)
• Chloroform (#05–3400, Sigma-Aldrich, USA)
• Medium-chain triglyceride oil (#02117401, Nisshin OilliO Group, Japan)

Equipment:

• Water bath (#TB-1 N, AS ONE, Japan)

Step-by-step protocol:

1. Stock solutions of 10 mM DOPC and 1 mM PI(4,5)P_2_ in chloroform were prepared by dissolving the lipid powders thoroughly and aliquoting them into appropriate volumes.
2. The aliquoted lipid solutions were stored at −20 °C until use. Degradation of the lipid hydrophilic headgroups or hydrophobic tails may impair the formation of a stable interfacial layer between the aqueous and oil phases.
3. To prepare the lipid film, 94 µL of DOPC and 60 µL of PI(4,5)P_2_ stock solutions were mixed in a 1.5 mL tube. The chloroform was evaporated using an air blower to form a dry lipid film on the inner surface of the tube.
4. The lipid film was re-dissolved in 1 mL of medium-chain triglyceride (MCT) oil. The final concentrations of DOPC and PI(4,5)P_2_ in the lipid-in-oil mixture were 0.94 mM and 0.06 mM, respectively.
5. To ensure complete dissolution of the phospholipids, the mixture was incubated in a water bath at 60 °C for 3 h and mixed by inversion. Extended heating of the oil phase (e.g., at 80 °C overnight) may cause degradation of the MCT oil and the phospholipid. If the oil phase turns brown or yellow, it should not be used in the experiments.
6. The prepared lipid-in-oil mixture was stored at room temperature until use. Storing the oil phase in a freezer may lead to condensation, resulting in the risk of contamination by water. Furthermore, the oil phase should be used within one month to avoid degradation of the phospholipids.

## Preparation of the glass flow chamber

**Fig. 2:**
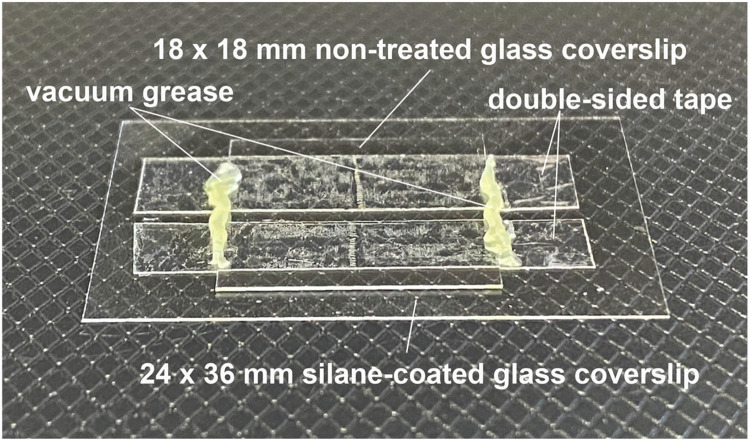
**Silane-coated glass flow chamber.** To construct the chamber, strips of double-sided tape were placed on a silane-coated glass coverslip at 1-mm intervals to serve as spacers. A non-treated glass coverslip was then placed on top, and the areas in contact with the tape were lightly pressed to form the flow chamber. The resulting flow chamber had a volume of approximately 3 μL.

Note: As the flow chamber is single-use, the absence of fluid flow and stability of the W/O droplets should be confirmed during the assay.

The *in-droplet* actin filament gliding assay was performed using a small-volume glass flow chamber. The glass chamber was assembled by placing a small non-treated glass coverslips (18 × 18 mm) on top of a silane-coated glass coverslips (24 × 36 mm), using double-sided tape as a spacer (Fig. 2). After infusing the emulsion containing W/O droplets (described in a later section), both ends of the flow path were sealed with vacuum grease. Fluid flow inside the glass chamber can obstruct observation. Therefore, it is important to ensure that no gaps are formed between the glass coverslips, the double-sided tape, and the vacuum grease after sealing the chamber.

Materials:

• 24 mm × 36 mm silane-coated glass coverslips, prepared as described in the previous section
• 18 mm × 18 mm glass coverslips (#C218181, Matsunami Glass Industry, Japan)
• Double-sided tape (#W-12, Scotch 3 M, USA)
• Vacuum grease (#60700, M&I Materials, UK)

## Preparation of the aqueous phase for W/O droplets

An aqueous phase containing biomolecules is prepared for encapsulation in W/O droplets. It is recommended to prepare the aqueous phase immediately before each experiment because its stability is limited to a few tens of minutes. During storage on ice, care should be taken to avoid prolonged standing or repeated thawing, which may affect the activity of the biomolecule. Although a single preparation can be used for multiple W/O droplet constructions within its stable period, it should not be used beyond this period.

Proteins:

• 2.7 μM myosin ID (stored at −80 °C)
• 123 μM calmodulin (stored at −80 °C)
• 60 μM G-actin (stored in liquid nitrogen)

Chemicals and Reagents:

• 1 M Sucrose (#30404–45, Nacalai Tesque, Japan)
• 1 M Glucose (#049–31165, Fujifilm Wako Pure Chemical, Japan)
• 100 mM ATP (#A7699, Sigma-Aldrich, USA)
• 132 µM Alexa488-phalloidin (#A12379, Thermo-Fisher, USA)
• ATP regeneration system (500 mM creatine phosphate (#10621714001, Roche, Switzerland) and 30 mg mL^⁻1^ creatine phosphokinase (#10127566001, Roche, Switzerland))
• Oxygen scavenging system (5 kU mL^⁻1^ glucose oxidase (#G2133, Sigma-Aldrich, USA) and 4 mg mL^-1^ catalase (#C3155, Sigma-Aldrich, USA))

Buffer:

• AT buffer (50 mM Tris–HCl, pH 7.4, 25 mM KCl, 1 mM MgCl_2_, and 1 mM EGTA)

Step-by-step protocol:

1. Myosin ID, G-actin, and calmodulin stored at −80 °C or in liquid nitrogen were thawed immediately by hand and kept on ice.
2. A 20 µL aqueous phase was prepared by supplementing AT buffer with the following components:

• 360 mM sucrose
• 100 mM glucose
• 1 mM ATP
• 0.7 μM myosin ID
• 1.5 μM calmodulin
• 1.2 μM G-actin
• 2.6 µM Alexa488-phalloidin
• ATP regeneration system (e.g., 5 mM creatine phosphate and 0.3 mg mL^⁻1^ creatine phosphokinase)
• Oxygen scavenging system (e.g., 50 U mL^⁻1^ glucose oxidase, 40 µg mL^-1^ catalase)

3. The aqueous phase was temporarily stored on ice until use and can remain stable for several hours, allowing a single preparation of the aqueous phase to be used for multiple W/O droplet preparations.

## W/O droplet formation and introduction into flow chamber

The W/O droplet is spontaneously formed as the aqueous phase is separated from the oil phase by a phospholipid membrane. G-actin monomers contained within the aqueous phase polymerize to form actin filaments. Myosin ID interacts with actin filaments via its N-terminal motor head domain and with PI(4,5)P_2_ in the lipid membrane via its C-terminal pH domain. The gliding motion of the actin filaments is induced by membrane-bound myosin ID.

Chemicals and Reagents:

• Aqueous phase (360 mM sucrose, 100 mM glucose, 1 mM ATP, 0.7 µM myosin ID, 1.5 µM calmodulin, 1.2 µM G-actin, 2.6 µM Alexa488-phalloidin, ATP regeneration system, and oxygen scavenging system in AT buffer)
• Lipid-in-oil mixture (0.94 mM DOPC and 0.06 mM PI(4,5)P_2_ in MCT oil)

Equipment:

• Metal tube rack

Step-by-step protocol:

1. Add 3 µL of the aqueous phase and 20 µL of the lipid-in-oil mixture into a 600 µL tube.
2. To ensure sufficient mixing of the aqueous and oil phases, vigorously rub the tube against a metal tube rack four times (Fig. 3a). This procedure allows droplets of the aqueous phase containing actin and myosin ID to disperse into the oil phase. The phospholipids within the oil phase subsequently form a monolayer at the oil–water interface, resulting in the generation of stable W/O droplets.

**Fig. 3:**
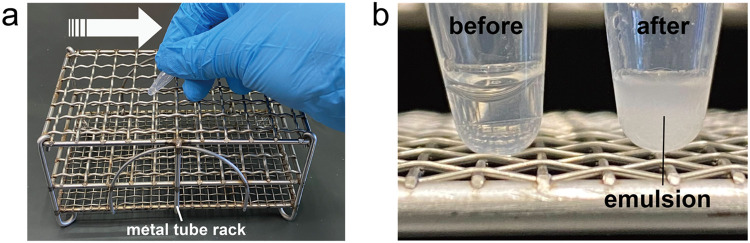
**Construction of emulsion containing W/O droplet.** (**a**) To construct W/O droplets, the 600 µL tube containing the aqueous phase and lipid-in-oil mixture was vigorously rubbed against a metal tube rack in the direction indicated by the white arrow. (**b**) Tube containing the aqueous phase and lipid-in-oil mixture before rubbing (left) and tube containing the emulsion after rubbing (right). Within the tube, the aqueous phase and lipid-in-oil mixture were mixed, resulting in the formation of W/O droplets.
3. After rubbing, confirm that the liquid in the tube appears cloudy, indicating the formation of an emulsion containing W/O droplets (Fig. 3b).
4. Introduce 3 µL of the emulsion containing the W/O droplets into the glass flow chamber.
5. Seal the glass flow chamber with vacuum grease.

Validation:

The successful formation of W/O droplets can be confirmed by the appearance of a cloudy emulsion in the tube after vigorous mixing (Fig. 3b). This visual check ensures that the aqueous phase has been properly dispersed into the oil phase and that phospholipid monolayers have formed at the droplet interfaces.  

Troubleshooting:

- If the mixture of oil and aqueous phases does not appear cloudy after rubbing and separates again into distinct phases, mixing is insufficient. In this case, additional rubbing is required.
- If the phases separate even after additional mixing, repeat the experiments using a fresh lipid-in-oil mixture because the original mixture may have degraded.

## Fluorescence imaging of *in-droplet* actin filament gliding assay

**Fig. 4:**
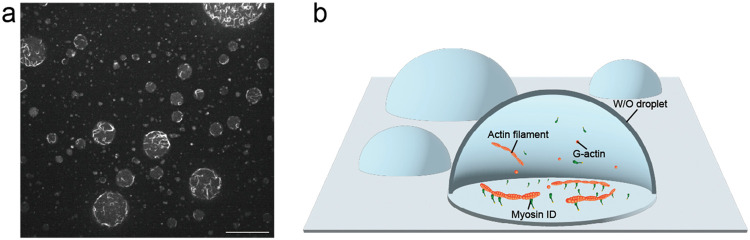
**Image of *in-droplet* actin filament gliding assay.** (**a**) A field of view of the *in-droplet* actin filament gliding assay. The aqueous phase of W/O droplets contained numerous actin filaments formed by the polymerization of G-actin monomers. Actin filaments were located at the inner surface of the W/O droplet via membrane-bound myosin ID. Most actin filaments successfully exhibited gliding motion induced by myosin ID on the inner surface of the W/O droplets. Scale bar, 20 µm. (**b**) Schematic of the *in-droplet* actin filament gliding assay.

Images of *in-droplet* actin filament gliding assay were acquired using a microscope (IX83, Evident, Japan) with objective lens (UPLSAPO60XW 1.2 NA, Evident, Japan) equipped with an sCMOS camera (ORCA-fusion C14440−20UP, Hamamatsu Photonics, Japan), spinning disk confocal unit (CSU-X1, Yokogawa Electric, Japan), a stable stage (KS-N, Chuukousha Seisakujo, Japan), a pulse motor (SGSP-13ACT, Sigma Koki, Japan), and a controller (QT-CM2, Chuo Precision, Japan). Alexa 488-labeled actin filaments were illuminated with 488 nm laser light source (SAPPHIRE 488−20 CW CDRH, Coherent, USA). Image acquisition was controlled by Micro-Manager software. Images were acquired with 1 s exposure time (Fig. 4a, b) (Movie 1).

Troubleshooting:

- If actin filaments show rapid photobleaching or their gliding velocity gradually decreases, check that the illumination intensity is not excessively high and confirm that the aqueous phase contains an oxygen scavenging system and an ATP regeneration system.
- If too many actin filaments are observed in the droplet, reduce the amount of G-actin in the aqueous phase.
- If actin filaments frequently bind to and dissociate from the inner surface of the W/O droplet, the myosin concentration should be increased.
- If the gliding motion of most actin filaments within a single W/O droplet stalls simultaneously, the silanization of the glass coverslips may have failed.

## Data analysis

**Fig. 5:**
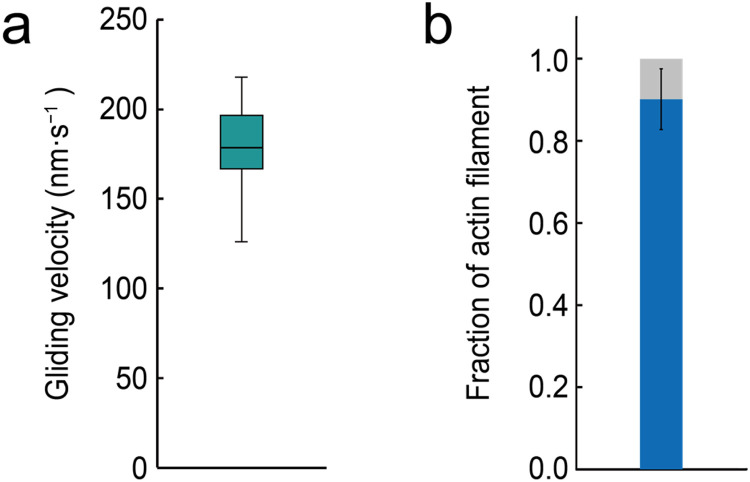
**Quantification of gliding actin filament velocity.** (**a**) Box plot showing the gliding velocity of actin filaments driven by myosin ID. The box represents the 25th–75th percentiles, and the mean value is indicated by a black line. The mean gliding velocity was 178 ± 26 nm·s^−1^ (mean ± standard deviation; *n* = 32 actin filaments). (**b**) Bar graph showing the fraction of gliding (blue) and stalled (grey) actin filaments on the inner surface of W/O droplets. The mean fraction of gliding actin filaments was 0.9 ± 0.1 (mean ± standard deviation; *n* = 6 droplets).

Gliding actin filaments on the inner surface of the W/O droplet were manually tracked using Mark 2.6 software with a two-dimensional Gaussian fitting algorithm [17]. The *x-* and *y-*coordinates of the leading edge of each gliding actin filament were manually traced and exported for each frame. The actin filament gliding velocities were calculated by linear fitting of five consecutive data points from the time−distance (*xy*) plots (Fig. 5).

## Limitations

The *in-droplet* actin filament gliding assay enables quantitative analysis of actomyosin dynamics on the membrane surface within W/O droplets. However, it reproduces only certain aspects of the cellular environment, and therefore provides limited insight into overall cell function, even when focusing on the interaction between myosin ID and lipids. Furthermore, other cellular factors such as complex cytoskeletal structures, molecular crowding, and regulatory proteins are not represented in this system. In addition, observations are currently restricted to the two-dimensional plane at the bottom of the droplets, making it difficult to measure actomyosin movement on curved membrane surfaces. Future developments enabling three-dimensional observation could allow detailed evaluation of actomyosin motility on membranes with curvature. Despite these limitations, this assay provides a valuable platform for studying the fundamental properties of membrane-bound myosins under controlled conditions.

## CRediT authorship contribution statement

**Yusei Sato:** Conceptualization, Methodology, Investigation, Formal analysis, Writing – original draft. **Rieko Sumiyoshi:** Software, Formal analysis. **Masahito Hayashi:** Methodology. **Masahiko Yamagishi:** Supervision, Resources. **Junichiro Yajima:** Supervision, Conceptualization, Writing – original draft.

## Declaration of competing interest

The authors declare that they have no known competing financial interests or personal relationships that could have appeared to influence the work reported in this paper.

## Data Availability

Data will be made available on request.

## Appendix Supplementary materials

Image, application 1
