# Supplementary material for: A method for reconstituting the motility of membrane-bound myosin on the surface of the cell-sized W/O droplet
Source: MethodsX. 2025 Dec 9;16:103755. doi: 10.1016/j.mex.2025.103755 (PMC12771316; doi:10.1016/j.mex.2025.103755)
Supplement: Supplementary file 1 [file mmc1.zip › mmc2.docx]

**Movie 1** ***In-droplet* actin filament gliding assay.** Gliding actin filaments driven by myosin ID molecules on the inner surface of W/O droplets containing 6% PI(4,5)P_2_. This movie shows Alexa 488-labeled gliding actin filaments (100× actual speed). Scale bar: 20 μm. Timestamp: min:s.
